# *Tubb4b* is required for multi-ciliogenesis in the mouse

**DOI:** 10.1242/dev.201819

**Published:** 2024-01-04

**Authors:** Mycah T. Sewell, Emilie Legué, Karel F. Liem

**Affiliations:** ^1^ Department of Genetics, Yale University School of Medicine, New Haven, CT 06520, USA; ^2^ Vertebrate Developmental Biology Program, Department of Pediatrics, Yale University School of Medicine, New Haven, CT 06520, USA

**Keywords:** Tubulin, Cilia, Axoneme, Mouse, Tubulinopathy, Ciliopathy, Ciliary microtubules, Tubulin code

## Abstract

Cilia are microtubule (MT)-based organelles present on the surface of nearly all vertebrate cells. MTs are polymers of α- and β-tubulins that are each encoded by multiple, individual isotype genes. Tubulin isotype composition is thought to influence MT behaviors. Ciliary MTs differ from other MTs in the cell in terms of organization, stability and post-translational modifications. However, little is known about the tubulin isotypes that build ciliary MTs and the functional requirements for tubulin isotypes in cilia have not been examined in vertebrates. Here, we have tested the role of the β-tubulin isotype genes in the mouse that harbor a conserved amino acid motif associated with ciliated organisms. We found that Tubb4b localizes to cilia in multi-ciliated cells (MCCs) specifically. In respiratory and oviduct MCCs, Tubb4b is asymmetrically localized within multi-cilia, indicating that the tubulin isotype composition changes along the length of the ciliary axonemal MTs. Deletion of *Tubb4b* resulted in striking structural defects within the axonemes of multi-cilia, without affecting primary cilia. These studies show that *Tubb4b* is essential for the formation of a specific MT-based subcellular organelle and sheds light on the requirements of tubulin isotypes in cilia.

## Teaser

**Summary:** *Tubb4b* knockout mice reveal structural defects within the axonemes of multi-cilia.

## INTRODUCTION

Microtubules (MTs) are fundamental cytoskeletal components present in all eukaryotic cells and essential for functions such as cell division, cell motility and intracellular transport. MTs comprise polymers of α- and β-tubulin heterodimers, with each class of tubulin encoded by multiple separate and highly conserved isotype genes (Luduena, 1998). The tubulin isotypes differ mainly in their C-terminal amino acid sequences, which are the site of numerous post-translational modifications that have been shown to influence MT function (Chakraborti et al., 2016; Janke and Magiera, 2020; Sirajuddin et al., 2014). Several studies have shown that altering the tubulin isotype proteins that make up MTs changes MT properties both *in vivo* and *in vitro*. For example, molecular studies have established that MT stability, protofilament number and dynamics are regulated in part by β-tubulin isotype composition (Honda et al., 2017; Raff et al., 1997; Savage et al., 1989; Ti et al., 2018). These studies are consistent with the idea of a ‘tubulin code’, which postulates that cells build MTs composed of distinct tubulin isotype combinations that, along with post-translational modifications, underlie MT functional diversity within the cell (Janke, 2014; Roll-Mecak, 2020; Verhey and Gaertig, 2007).

Cilia are microtubule-based organelles present on the cell surface of nearly all cells in the body. The MTs that form the cytoskeleton of cilia are highly organized and are arranged in nine doublets in primary cilia with an additional central pair of MT singlets in motile cilia. Unlike cellular MTs, which are highly dynamic and undergo rapid growth and collapse (Mitchison and Kirschner, 1984), ciliary MTs are stable and anchored at their minus end by their association with basal bodies. Ciliary MTs harbor a distinct array of post-translational modifications, including acetylation and polyglutamylation (Wloga et al., 2017). Although ciliary MT have distinct properties, it is unclear whether they require specific tubulin isotype proteins. Genetic experiments analyzing the flagella of sperm tails in *Drosophila* revealed that swapping tubulin isotypes resulted in striking MT defects (Hoyle and Raff, 1990), indicating that the specific tubulin isotype found in sperm flagella cannot be substituted (Raff et al., 1997, 2000). These functional studies, along with sequence analyses of tubulins in ciliated organisms led to the hypothesis that a subset of β-tubulins that harbor an evolutionarily conserved C-terminal motif (EGEFXXX, where X is D or E) are crucial for cilia and the central pair (Dutcher, 2001; Nielsen et al., 2001; Raff et al., 1997). Aside from a recent preprint reporting the consequences of *Tubb4b* mutation in mouse (Mechaussier et al., 2022 preprint), genetic studies testing the roles of tubulin isotypes within cilia in mammals that express eight β-tubulin isotype genes have not been performed.

Here, we have tested the requirements of the only two β-tubulins that harbor the putative C-terminal ciliary motif in mouse: *Tubb4a* and *Tubb4b*. We show that Tubb4 protein is localized to cilia in multiple lineages of multi-ciliated cells (MCCs) – specialized post-mitotic cell types that project dozens of motile cilia that drive directed fluid flow in select organs. Deletion of *Tubb4b* resulted in striking structural defects in the axonemal component of multi-cilia, whereas *Tubb4a* deletion did not disrupt cilia. Neither tubulin was required for primary cilia. These studies demonstrate that tubulin isotype composition differs in the MTs of different types of cilia. The deletion of a single tubulin isotype protein, Tubb4b, is sufficient to disrupt cilia in MCCs.

## RESULTS AND DISCUSSION

**Fig. 1.**
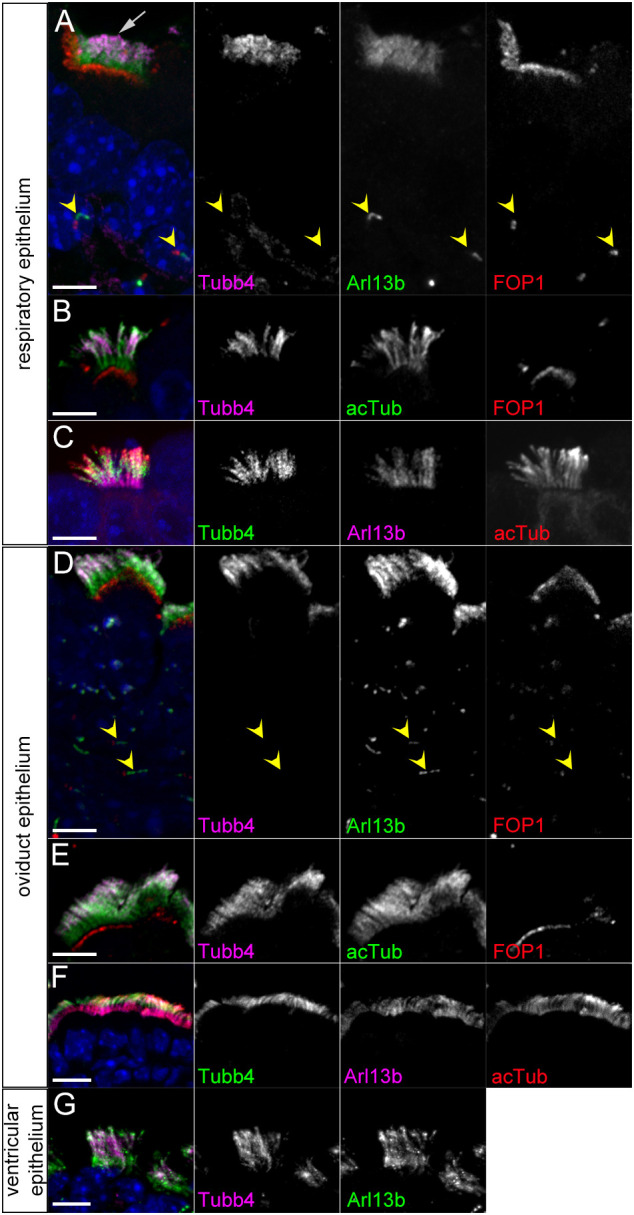
**Tubb4 immunofluorescence staining of multi-ciliated cells.** (A-C) Immunofluorescent staining of respiratory MCCs at (A) P1 and (B,C) P5. Tubb4 signal is enriched in distal-most 57±10.7% of axonemes (mean±s.d., *n*=85 cells from six animals). (D-F) Immunofluorescent staining of oviduct epithelium in adults. Tubb4 signal is enriched in distal 54.4±9.8% of axonemes (*n*=36 cells from two animals). Scale bars: 5 µm in A-E; 10 µm in F. (G) Tubb4 staining of adult ependymal cells. Scale bar: 10 µm. Arrow indicates MCC cilia; arrowheads indicate primary cilia.

To characterize the expression of Tubb4a and Tubb4b, we performed immunofluorescence staining on sections of murine tissue using a Tubb4-isotype specific antibody (Banerjee et al., 1992). We found strong staining in ciliary axonemes of MCCs in all tissues examined. In the respiratory tract of early post-natal mice, Tubb4 staining was enriched in ciliary axonemes of epithelial MCCs (Fig. 1A-C). Within the multi-cilia, Tubb4 staining was overlapping but distinct from the immunofluorescent staining of two commonly used cilia markers: Arl13b, a small G-protein; and acetylated α-tubulin (acTub), a post-translational modification present on alpha tubulin isotype proteins. Arl13b and acTub uniformly labeled the proximal-distal length of cilia axonemes. In contrast, Tubb4 staining was not uniform in multi-cilia and was weaker in the proximal region of the cilia abutting the basal bodies labeled by FOP1 compared with the rest of the axoneme (Fig. 1A-C). We measured Tubb4 staining intensity along the Arl13b-labelled cilia and found that the Tubb4 was most intense in the middle of the axoneme, tapering towards the distal end, while very weak in the proximal cilium (Fig. S1). Notably, the primary cilia that were detected by Arl13b staining on cells basal to the MCCs did not show Tubb4 staining (Fig. 1A).

In the oviduct, Tubb4 also labeled multi-cilia in a similar pattern to that of the MCCs in the respiratory tract, with weak staining in the proximal cilium (Fig. 1D-F) and Tubb4 staining was absent in primary cilia (Fig. 1D). We also observed strong Tubb4 staining in the multi-cilia of the ependymal cells that line the lateral ventricles in the CNS (Fig. 1G). Unlike in respiratory and oviduct MCCs, Tubb4 staining within the ependymal cell axonemes showed uniform staining, with Tubb4 present along the entire ciliary axoneme. Together, these results indicate that Tubb4 localized to multi-cilia in a variety of tissues in the mouse. These results also show that tubulin isotype composition differs between multi and primary ciliary MTs. Interestingly, the data also indicate that tubulin isotype composition can change along the length of ciliary MTs, raising the possibility that individual MTs could have different functional properties along its length.

**Fig. 2.**
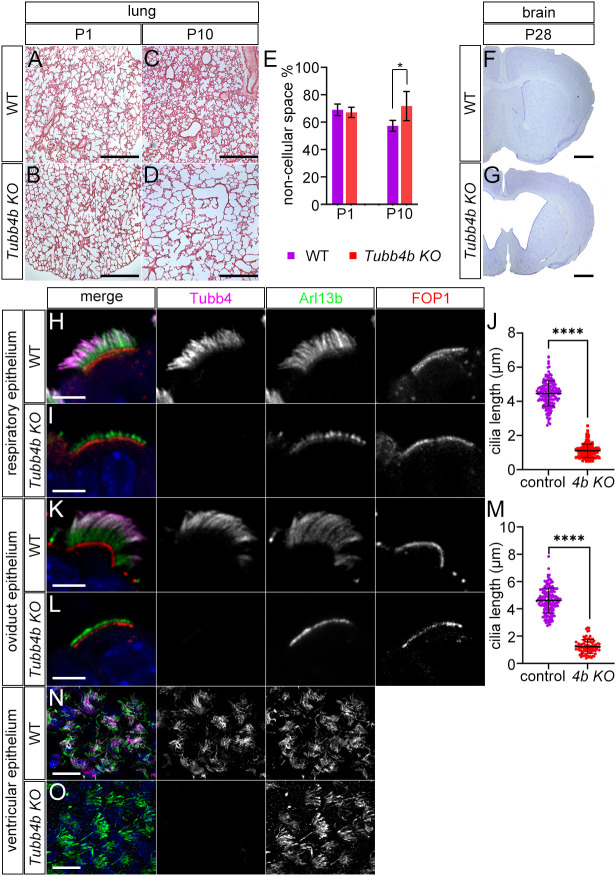
**Analysis of *Tubb4b* deletion.** (A-D) Hematoxylin and Eosin (H&E) staining of wild-type and *Tubb4b* KO lung sections at postnatal days 1 and 10. Scale bars: 500 µm. (E) Percentage of non-cellular space in H&E sections of lungs (four *Tubb4b* KO and three wild-type P1 animals, and four *Tubb4b* KO and three wild-type P10-P12 animals were analyzed). *P=0.0436 unpaired *t*-test. (F,G) Nissl staining of coronal sections through wild-type and *Tubb4b* KO forebrains at P28. Scale bars: 1 mm. (H,I) Immunofluorescence staining of control respiratory MCC and *Tubb4b* KO MCC at P1. Scale bars: 5 µm. (J) Length of cilia in respiratory *Tubb4b* KO MCC based on Arl13b staining (1.1±0.4 μm, *n*>130) and control respiratory MCC (4.6±0.8 µm, *n*>150 cells) from three animals/genotype. ****P<0.0001 unpaired *t*-test. (K,L) Immunofluorescence staining of control and *Tubb4b* KO oviduct sections (adult). Scale bars: 5 µm. (M) Length of oviduct MCC cilia based on Arl13b staining: *Tubb4b* KO=1.2±0.6 µm, *n*=86 from two animals; wild type=4.4±1 µm, *n*>150 cells from 3 animals. ****P<0.0001 unpaired *t*-test. (N,O) Immunofluorescence staining of apical surface of the lateral ventricle of wild-type and *Tubb4b* KO P14 animals showing ependymal cells observed *en face*. Scale bars: 10 µm.

We next analyzed a *Tubb4b* knockout to test the role of *Tubb4b* in cilia, because we previously reported that the *Tubb4a* knockout mouse was non-phenotypic (Fertuzinhos et al., 2022). We generated *Tubb4b* knockout mice from EUCOMM embryonic stem (ES) cells, in which the middle two of the four exons in *Tubb4b* are deleted, resulting in an allele that is likely null and not transcribed, as shown by RT-PCR (Fig. S2). At birth, homozygous *Tubb4b* mutant mice appeared indistinguishable from wild-type or heterozygous controls. Litters genotyped between P0 and P3 displayed expected mendelian ratios (Table S1). Thereafter, homozygous mutants appeared smaller than wild-type littermates, and litters examined at P4-P6 and P7-P10 contained only 13% and 11% homozygous mutants, respectively, indicating increased mortality starting around P4-P6. Among homozygous mutant animals, >45% of pups died before P13 and >88% of pups did not survive to P21. We performed Hematoxylin and Eosin staining on lung tissue and found that *Tubb4b* mutants and controls presented similar lung histology at P1, with fine tissue arborization and alveoli (Fig. 2A,B). However, *Tubb4b* mutants soon developed histological changes in the lungs, and mutants analyzed at P10-P12 showed less fine tissue arborization in the small airways, resulting in an increase in non-cellular space in the lung sections (Fig. 2B,D,E). Nissl staining of coronal sections of the brain revealed that >80% of pups examined after P21 developed hydrocephalus (*n*=19) (Fig. 2F,G). These results indicated that *Tubb4b* is required in the mouse, with mutants developing histological changes within the lungs and increased mortality accompanied by more slowly developing hydrocephalus.

We next determined the cellular effects of *Tubb4b* deletion. We first analyzed MCCs of the respiratory tract in lung sections and found that homozygous deletion of *Tubb4b* resulted in a severe truncation of multi-cilia. Arl13b staining of *Tubb4b* mutant respiratory MCC showed only short stubs (∼1 µm) of Arl13b staining adjacent to the basal bodies (Fig. 2H-J; Fig. S3). Similar results were observed in the oviduct MCCs as deletion of *Tubb4b* disrupted multi-cilia in which, again, only short remnants of cilia identified by Arl13b staining were present adjacent to the basal bodies (Fig. 2K-M). *Tubb4b* deletion did not appear to disrupt basal bodies as they were present at the apical surface of the cells, which is similar to controls, as determined by FOP1 staining. Loss of *Tubb4b* did not result in shortened multi-cilia in ependymal cells, as Arl13b staining showed cilia of similar length and morphology to controls (Fig. 2N,O). The Tubb4 signal was not detected in *Tubb4b* mutants, identifying the Tubb4 ciliary staining as Tubb4b protein. *Tubb4a* KO mice did not show ciliary defects and Tubb4 staining was present similar to that in wild type, indicating that Tubb4b was the tubulin isotype present in MCC cilia (Fig. S4).

**Fig. 3.**
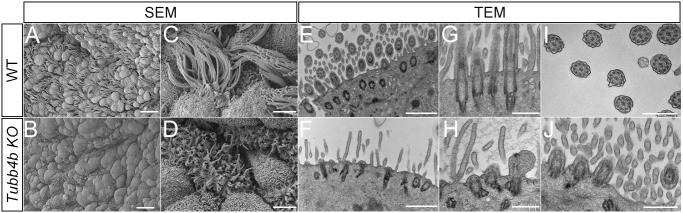
**Electron microscopy of *Tubb4b* KO respiratory MCC.** (A-D) Scanning electron microscopy of apical surface of respiratory epithelium. (A,C) Wild-type and (B,D) *Tubb4b* KO respiratory epithelium at P2. Scale bars: 10 µm in A,B; 2 µm in C,D. (E-J) Transmission electron microscopy of wild-type and *Tubb4b* KO respiratory epithelium. Scale bars: 1 µm in E,F; 500 nm in G-J.

We further visualized the cilia defects using electron microscopy (EM). Scanning EM of the surface of the wild-type respiratory epithelium of the trachea showed many MCCs characterized by the numerous long cilia extending from the apical surface, interspersed among the respiratory epithelial cell types (Fig. 3A,C). In *Tubb4b* mutants, MCCs were similarly present; however, they only showed very few short cilia (Fig. 3B,D), confirming our immunofluorescence analysis. To determine the basis of the ciliary defects, we examined respiratory MCCs using transmission EM (Fig. 3E-J). In wild-type animals, we detected robust cilia emanating from basal bodies extending from the apical surface of MCCs. These cilia contained the classic 9+2 configuration of MTs associated with motile cilia (Fig. 3I). *Tubb4b* mutants similarly showed basal bodies docked at the cell surface; however, many failed to show an extension of the ciliary axoneme (Fig. 3F) or showed deformed axonemes (Fig. 3H). We were unable to identify cross-sections of ciliary axonemes showing microtubule doublets with a central pair, but we found cross-sections of cilia a short distance from the cell surface showing the distal transition zone (basal plate): the region of the cilium proximal to the axoneme in respiratory multi-cilia (Fig. 3J). These results indicate that *Tubb4b* is required for the axonemes in respiratory MCCs. Owing to the severity of the structural defect, it was not possible to determine whether the central pair was specifically affected in *Tubb4b* mutants.

**Fig. 4.**
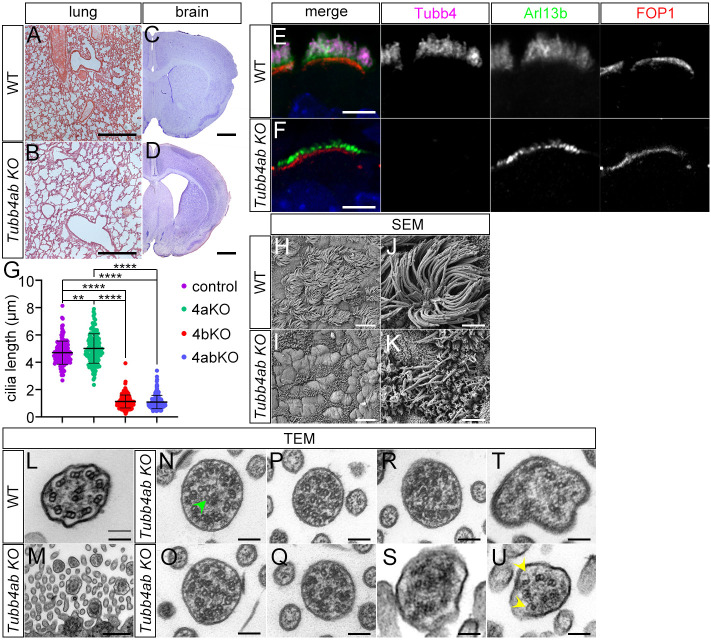
***Tubb4a/Tubb4b* double-mutant analysis.** (A,B) Hematoxylin and Eosin (H&E) staining of wild-type and *Tubb4a/Tubb4b* double KO (*Tubb4ab* KO) lung sections at postnatal day 10. Scale bars: 500 µm. (C,D) Nissl staining of coronal sections through wild-type and *Tubb4ab* KO forebrains (adult). Scale bars: 1 mm. (E,F) Immunofluorescence staining of wild-type and *Tubb4ab* KO respiratory MCC at P1. Scale bars: 5 µm. (G) Cilia lengths of control, *Tubb4a* KO (4aKO), *Tubb4b* KO (4bKO) and *Tubb4ab* KO (4abKO) respiratory MCC cilia. Control=4.7±0.85 µm; 4aKO=5.01±1.2 µm; 4bKO*=*1.13±0.47 µm; 4abKO=1.09±0.55 µm. Over 140 cells were analyzed from three animals/genotype. ****P<0.0001, **P=0.002 one-way ANOVA followed by Tukey's post-hoc test. (H-K) Scanning electron microscopy of the apical surface of wild-type and *Tubb4ab* KO respiratory epithelium at P11. (L-Q) Transmission electron microscopy of (L) wild-type and (M-U) *Tubb4ab* KO respiratory MCCs at (N-R,T) P2 and (L,M,S,U) P11. Scale bars: 10 µm in H,I; 2 µm in J,K; 100 nm in L,N-U; 500 nm in M. Arrowheads indicate a central pair in N and open B-tubules in U.

The mouse genome contains two β-tubulin isotypes that harbor the C-terminal cilia motif: *Tubb4b* and *Tubb4a*. It was therefore possible that *Tubb4a* could partially compensate for the loss of *Tubb4b* in the *Tubb4b* KO mice*.* To test this hypothesis, we generated *Tubb4a*;*Tubb4b* double mutant mice and analyzed multi-cilia. Double mutant mice were born and followed a phenotypic course that closely resembled the *Tubb4b* KO single mutants: they developed similar histological changes in the small airways of the respiratory tract at P10-P12 (Fig. 4A,B) as well as hydrocephalus (Fig. 4C,D). Multi-cilia in respiratory MCCs were severely truncated, similar to *Tubb4b* single mutants with a short stub of Arl13b staining observed adjacent to the basal bodies (Fig. 4E-G). Unexpectedly, although severely shortened, occasional longer cilia were observed emanating from the cell surface in the *Tubb4a;Tubb4b* double KO mutants using scanning electron microscopy (SEM) (Fig. 4H-K). Whereas wild-type cilia showed the 9+2 microtubule axonemal organization (Fig. 4L), transmission electron microscopy (TEM) analysis of double mutant cilia showed structural defects. None of cross-sections through mutant axonemes showed the nine symmetric MT doublets surrounding a central pair, which is characteristic of wild-type axonemes (*n*=38). All axonemes observed showed the presence of singlets (Fig. 4N-U) and disorganization of the nine symmetric doublets (Fig. 4N-P) (38/38). We observed occasional dysmorphic cilia (Fig. 4S,T) (2/38) and cilia with MTs with abnormal open B-tubules at the outer junction (Fig. 4U) (2/38). Although double mutant cilia had severe structural defects, we were able to identify cilia that appear to contain the central pair, indicating that *Tubb4a* and *Tubb4b* were not strictly required for this structure (Fig. 4N). The presence of axonemal structures in the double mutants but not in *Tubb4b* single mutants is not understood, but we speculate that the additional deletion of *Tubb4a* could change the tubulin protein availability of the other tubulin isotypes or could alter combinatorial tubulin isotype composition to allow more axonemal development. However, the results did show that concomitant deletion of *Tubb4a* did not worsen the ciliary phenotypes of *Tubb4b*.

The basic cytoskeletal component of motile cilia consists of nine doublet MTs extending from the basal body into the ciliary axoneme surrounding a central pair. Mammalian cells express multiple α- and β-tubulin isotype proteins that are free to intermingle and assemble into heterodimers, and to incorporate into the microtubules of the cell (Lewis et al., 1987). Strikingly, we find that Tubb4b protein was not uniformly distributed in cilia in the same way as acTub in respiratory and oviductal MCCs. The mechanism by which asymmetric enrichment of Tubb4b is established is unclear. Protein translation does not occur within cilia and all proteins such as tubulins require transport into the cilium. Monomeric Tubb4b could be transported to the distal cilium, where it could accumulate and be incorporated into the MTs as part of a ciliary sub-compartment. A proximal axonemal sub-compartment has been previously described for the ciliary protein inversin in both motile and primary cilia, a region where novel fibril-substructures have been described (Bennett et al., 2020; Shiba et al., 2009). In addition, the dynein motor proteins DnaH11 and DnaH5 localize to the proximal and distal regions of respiratory multi-cilia, respectively, where they define distinct outer dynein arm complexes (Dougherty et al., 2016). Alternatively, a rapid increase in the availability of Tubb4b monomers during the extension or maintenance of the cilium could potentially establish the distal enrichment of Tubb4b in the axonemes, which could perdure due to the stability of MTs within cilia.

The distribution of Tubb4b protein within multi-cilia is cell-type specific, appearing uniform throughout the axoneme in ependymal cells but asymmetric in respiratory and oviductal multi-cilia. MTs in multi-cilia are subject to increased tortional stress when compared with MTs within the cell body or primary cilia, as multi-cilia are motile and beat to drive fluid flow. Interestingly, a previous study has shown that application of monoclonal antibodies against Tubb4 can block ciliary beating in cultured bovine tracheal cells, indicating that functional Tubb4b is required for cilia motility (Vent et al., 2005). The enrichment of Tubb4b distally is potentially functionally significant, as tubulin isotype composition regulates MT properties and behavior (Janke, 2014; Roll-Mecak, 2020). Previous studies have shown that MTs assembled from the Tubb4b and Tubb1 isotype proteins polymerized more rapidly and were less dynamic than MTs assembled with the Tubb3 isotype (Vemu et al., 2017). The observation that tubulin isotype composition can change from the minus to plus end within individual MTs raises the possibility that ciliary MTs might use the combinatorial diversity of tubulin to generate specialized MTs for multiciliary function (Roll-Mecak, 2020).

It has been unclear whether mammalian cells use specific tubulin isotype proteins to build or maintain different subcellular MTs. Here, we showed that deletion of *Tubb4b* resulted in striking axonemal defects in motile multi-cilia in the trachea and oviduct cells. Although ependymal cells were not structurally affected by *Tubb4b* deletion, mutant mice developed hydrocephalus. This phenotype could be caused by compromised cilia motility (Wallmeier et al., 2022), as ciliary motility was associated with Tubb4b function in cultured cells (Vent et al., 2005). *Tubb4b* deletion likely did not functionally impair all motile cilia, as we did not detect laterality defects in *Tubb4b* or *Tubb4a/Tubb4b* mutant animals, suggesting that single motile cilia of embryonic node cells were unaffected (Little and Norris, 2021). *Tubb4b* deletion could cause defects due to a paucity of tubulins available for the cell to form MTs in MCCs. However, tubulin levels are tightly regulated and deletion of tubulin genes have been shown to be compensated for by upregulation of related tubulin family members in differentiated cells (Bittermann et al., 2019; Fertuzinhos et al., 2022; Latremoliere et al., 2018). The cellular defects of *Tubb4b* deletion appeared axoneme specific: MCCs differentiated and migrated normally, and basal bodies were found docked to the cell surface. The concomitant deletion of *Tubb4a*, which could further reduce tubulin levels, did not result in more-severe defects, suggesting a more-complex relationship between tubulin isotype protein expression and multi-ciliogenesis. Our data are consistent with functional multi-cilia axonemes requiring MTs comprising a high percentage of the Tubb4b isotype.

Interestingly, the loss of *Tubb4b* caused defects in a specific subcellular organelle. Deletion of several of the β-tubulins in the mouse did not result in phenotypes, as they may act redundantly (Bittermann et al., 2019; Fertuzinhos et al., 2022). However, deletion of *Tubb1* was reported to have specific defects in megakaryocytes during platelet formation (Schwer et al., 2001). Dominant amino acid substitutions in *TUBB4B* are associated with Leber's congenital amaurosis, a retinal degenerative disease consistent with cilia dysfunction (Luscan et al., 2017). We examined retinae of *Tubb4b* KO mice but did not detect histological changes in the retina compared with controls (Fig. S5). This result could be due to the different nature of the mutations: dominant missense versus recessive loss of function in the knockout mice.

A classic study identified a cilia-associated motif present in the C-terminal region of a subset of β-tubulins and postulated its importance for cilia (Dutcher, 2001; Luduena, 1998; Nielsen et al., 2001; Raff et al., 1997). Here, we tested the requirement of the two β-tubulins in the mouse that harbor this motif using genetic methods. We show that tubulin isotypes harboring the putative ciliary motif are not required for primary cilia, but *Tubb4b* is specifically required for proper axonemes in multi-cilia. Our work, together with another study that reported similar phenotypes resulting from *Tubb4b* mutations in mouse (Mechaussier et al., 2022 preprint), may also be informative regarding the role of TUBB4B in ciliopathic and tubulinopathic diseases in patients.

## MATERIALS AND METHODS

### Mouse lines

Mice were generated from *Tubb4b^tm1a(EUCOMM)hmgu^* ES cells obtained from the International Knockout Mouse Consortium and bred with CAG-Cre mice (Sakai and Miyazaki, 1997) to delete exons 2 and 3 to generate a null allele (*Tubb4b* KO mice). *Tubb4a* KO has been previously described (Fertuzinhos et al., 2022). Mice were analyzed on the FVB background, except for mice used for retinal histology, which were analyzed on a mixed FVB and C57B6/J background, and were bred to exclude the *Pdeb^rd1^* mutation associated with FVB mice (Gimenez and Montoliu, 2001). The primers 5′-CATCAATGTATCTTATCATG-3′ and 5′-CCTTCTGTGTAGTGCCCCTT-3′ were used to genotype the mutant *Tubb4b* allele. The primers 5′-CCAACTGACTGCGGGTAGAT-3′ and 5′-CCACTCTGACCTGTAAGAGA-3′ were used to genotype the wild-type *Tubb4b* allele. For the *Pdeb^rd1^* mutation, the primers 5′-CATGTCGTACAGCCCCTCTC-3′ and 5′-AAGCTAGCTGCAGTAACGCCATTT-3′ were used to genotype the mutant, and 5′-CATGTCCTACAGCCCCTCTC-3′ and 5′-ACCATTTGCAAGGAAAGCAC were used to genotype the wild-type alleles (Gimenez and Montoliu, 2001). For the *Tubb4a* mutants, the primers 5′-ACCCCACTGGGACCTATCAT-3′ and 5′-CACGGCTCTGGGAACATAGT-3′ were used to genotype the wild-type *Tubb4a* allele, and 5′-AACAACAAAAGGAAAATCTATTCAC-3′ and 5′-AGGCCACCCAACTGACCT-3′ were used to genotype the mutant *Tubb4a* allele. All animal studies were performed under an approved Institutional Animals Care and Use Committee mouse protocol according to Yale University institutional guidelines.

### RT-PCR

Total RNA was isolated from E17.5 embryos using TRIzol (Invitrogen) reagent. cDNA was synthesized using SuperScriptIII (Invitrogen) using random hexamers. *Tubb4b* was amplified using the *Tubb4b* specific primers 5′-TGCACCTGCAGGCTGGGCAGT-3′ and 5′-TGTTGCCAATGAAGGTGGCC-3′, which are located in the first and last exons of *Tubb4b*, respectively (four exons in total). *Tubb5* was amplified using 5′-CCGTAGCCATGAGGGAAAT-3′ and 5′-ATGCCATGTTCATCGCTTATC-3′ primers.

### Histological analysis

Paraffin wax-embedded tissues were sectioned at 10 µm on a Leica RM225 microtome. Lung sections were stained with Hematoxylin and Eosin (H&E). Brain sections were stained with Nissl/Cresyl Violet. Images were captured on a Zeiss Axioskop upright microscope.

### Lung cellularity analysis

A region of H&E images taken at 10× magnification that were devoid of main bronchi or blood vessels were selected for analysis. At least three 1 mm^2^ fields were analyzed per animal. Pixels on mutant and control images were divided into black (cellular) or white (non-cellular) using the threshold function in ImageJ. Within the selected regions of interest, the total number of selected pixels was counted in the two groups: black/cellular pixels and white/non-cellular pixels. Pixels were counted using Photoshop. Ratios and percentages were displayed using GraphPad/Prism 9.4.1.

### Immunofluorescence

Mice were perfused (P5 and older) or immersion fixed (P1-P2) with 4% PFA and tissues transferred to 30% sucrose and frozen in OCT (TissueTek). Tissues were sectioned at 12 µm or 40 µm (brain) on a Leica cryostat (CM30505). Antibodies used in this study were mouse anti-acetylated α-Tubulin (Sigma T7451, 1:4000), rabbit anti-Arl13b (Caspary et al., 2007; 1:5000), mouse anti-Arl13b (NeuroMab, 75-287, 1:500), mouse anti-β Tubulin IV (Sigma T7941, 1:2000) and rabbit anti-FGF1OP (Proteintech 11343-1-AP, 1:500). Secondary antibodies from ThermoFisher were used at 1:500 dilution: donkey anti-rabbit Alexa488 (A21206), goat anti-rabbit Alexa594 (A11012), goat anti-mouse Alexa 594 (A-11005), goat anti-mouse IgG2b Alexa568 (A21144), goat anti-mouse Alexa633 (A21126), goat anti-rabbit Alexa633 (A21071) and goat anti-mouse IgG1 Alexa 488 (A21121). Goat anti-mouse IgG2a Cy5 (115-175-206, 1:500) and goat anti-rabbit IgG1 Fab (111-547-003, 1:250) were from Jackson Labs. Slides were mounted with 10% Mowiol 4-88 (Sigma 81381).

### Cilia lengths

Cilia lengths were determined by analysis of immunofluorescence-stained tissue sections with images acquired using a Zeiss LSM 880 Airyscan confocal microscope or a Zeiss Axiovert microscope with a Hamamatsu Orca Flash 4.0 camera. Images were processed using Zen software. Cilia length was determined by measuring the length of the Arl13b staining that marked the cilium adjacent to FOP1 using ImageJ software. The average of three measurements were plotted per MCC and at least 40 MCC were measured per animal (at least three animals/genotype). Results were displayed using GraphPad/Prism version 9.4.1.

### Tubb4 signal intensity within cilia

Tubb4 signal intensity within cilia was determined by analysis of immunofluorescence-stained tissue sections stained using Tubb4 and Arl13b antibodies. Images were acquired using a Zeiss LSM 880 Airyscan confocal or Axioskop microscope and processed using Zen software. Tubb4 immunofluorescence signal intensity was measured using ImageJ software. Selected images were displayed as separate channels converted into grayscale images. Cilia to be analyzed were selected using the line tool. The full length of cilia was determined by Arl13b along the axoneme. Cilia were analyzed using the intensity measuring function in ImageJ. This analysis produced graphs plotting immunofluorescence signal intensity along the sampled cilia. *x*-values from the ImageJ graphs were normalized to a scale of 0-100, where 0 was the proximal aspect of the Arl13b staining and 100 was distal extent of the Arl13b staining. Every fourth *x*-value in each normalized coordinate set was selected for graphing. The corresponding *y*-values were then normalized to a scale of 0-100, where 100 was the highest intensity value of Tubb4 and 0 was the lowest value, respectively, along the Arl13b staining domain. These normalized coordinate sets were then graphed using GraphPad Prism. Forty-one respiratory cilia from wild-type animals at P1 and P5 were analyzed, from at least 15 cells/animal. Analysis was displayed with GraphPad/Prism version 9.4.1.

### Transmission electron microscopy

Tracheal tissues were immersion fixed in 2.5% glutaraldehyde and 2% paraformaldehyde in 0.1 M sodium cacodylate buffer (pH 7.4) for 1 h and post fixed in 1% osmium tetroxide for 1 h. The sample was rinsed in buffer and en-bloc stained in aqueous 2% uranyl acetate for 1 h followed by rinsing in distilled water, dehydrated in an ethanol series of 50%, 70%, 95% and 100%. They were further dehydrated in 100% propylene oxide and then infiltrated with Embed 812 (Electron Microscopy Sciences) resin. The samples were placed in silicone molds and baked at 60°C for 24 h. Blocks were sectioned using a Leica UltraCut UC7. 60 nm sections were collected on formvar/carbon-coated grids and contrast stained using 2% uranyl acetate and lead citrate. The sections were imaged using an FEI Tecnai Biotwin TEM at 80 kV and images were taken using Morada CCD and iTEM (Olympus) software.

### Scanning electron microscopy

The tissue was fixed as for TEM to 100% ethanol dehydration. Samples were then transferred and dried using a Leica 300 critical point dryer with liquid carbon dioxide as transitional fluid. The samples were orientated and glued to aluminum stubs, then sputter coated with 5 nm platinum 80/palladium 20 using a Cressington 208HR. The samples were viewed and digital images acquired in Zeiss CrossBeam 550 between 1.5 and 2 kV at a working distance of 12-15 mm.

## Supplementary Material

10.1242/develop.201819_sup1 Supplementary information
