# Supplementary material for: Tubb4b is required for multi-ciliogenesis in the mouse
Source: Development. 2024 Jan 4;151(1):dev201819. doi: 10.1242/dev.201819 (PMC10820790; doi:10.1242/dev.201819)
Supplement: Supplementary information [file develop-151-201819-s1.pdf]

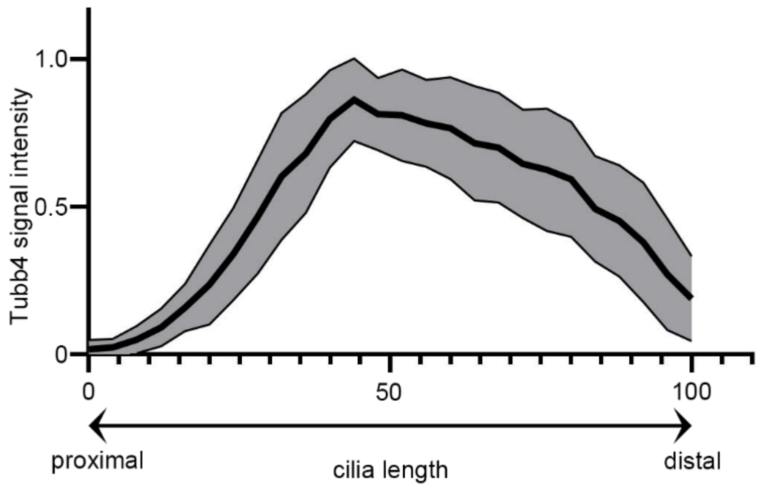

Fig. S1. Tubb4 immunostaining intensity along proximal-distal position of cilia
Quantification of Tubb4 immunostaining intensity along the proximal to distal length of respiratory MCC cilia. Fluorescent intensity average is depicted as a black line with one standard deviation displayed in gray.

Development • Supplementary information

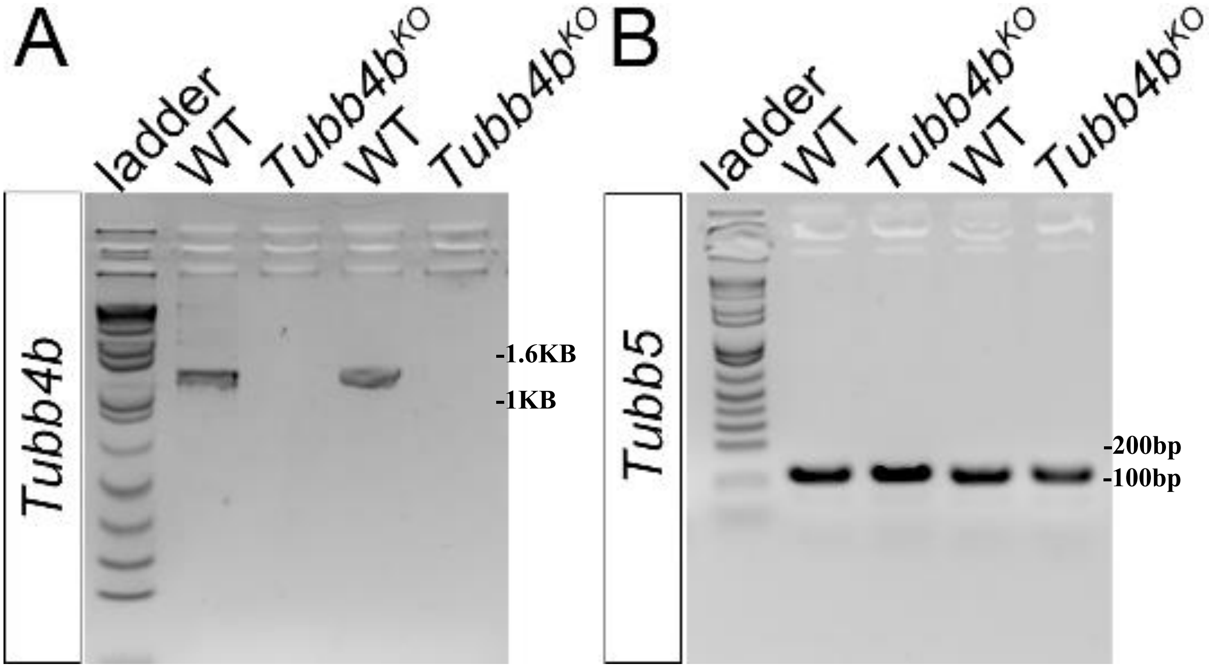

Fig. S2. RT-PCR of *Tubb4b* in mutants

(A) RT-PCR of *Tubb4b* homozygous mutant embryos and WT littermates using *Tubb4b* specific primers. Amplification of the predicted 1160 bp amplicon of *Tubb4b* was observed in the two control embryos but no bands were amplified from the two homozygous mutant embryos. (B) RT-PCR of identical *Tubb4b* homozygous mutant embryos and WT littermates using *Tubb5* specific primers. Amplification of the predicted 96 bp amplicon of *Tubb5* was observed in both control and *Tubb4b* embryos.

Development • Supplementary information

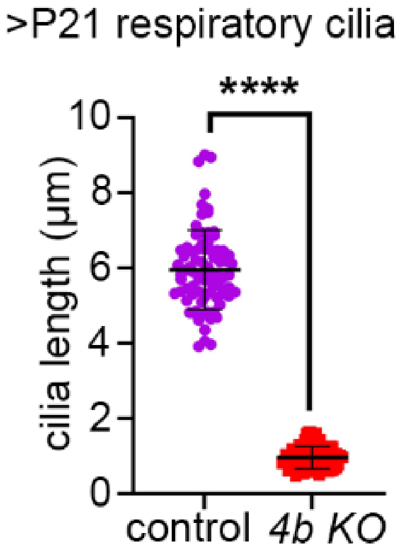

Fig. S3. Cilia measurements of *Tubb4b* mutant MCC in adult animals

Length of cilia in respiratory MCC in WT respiratory MCC (5.95±1.04µm, n=237 cilia from 79 MCC) and in *Tubb4b KO* (0.96±0.29µm, n=267 cilia from 89 MCC) as determined by Arl13b immunostaining. At least 25 cells from 3 animals/genotype were analyzed. ****p<0.0001 unpaired t-test. All animals were age >P21.

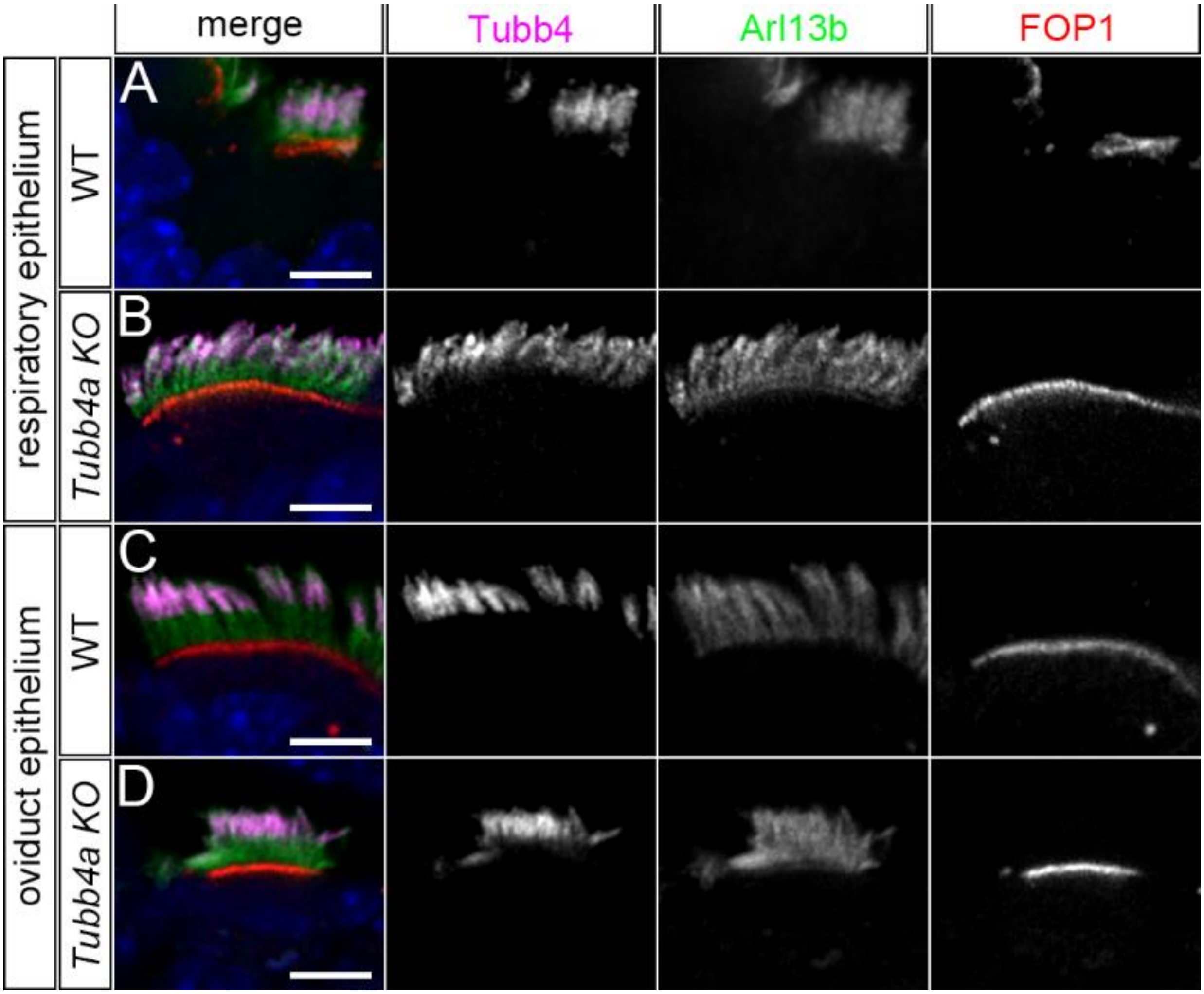

Fig. S4. *Tubb4aKO* respiratory epithelium. Immunofluorescence staining of (A) WT and (B) *Tubb4aKO* respiratory epithelium at P1. Scale=5µm. Immunofluorescence staining of (C) WT and (D) *Tubb4aKO* oviduct epithelium (adult). Scale=5µm.

Development • Supplementary information

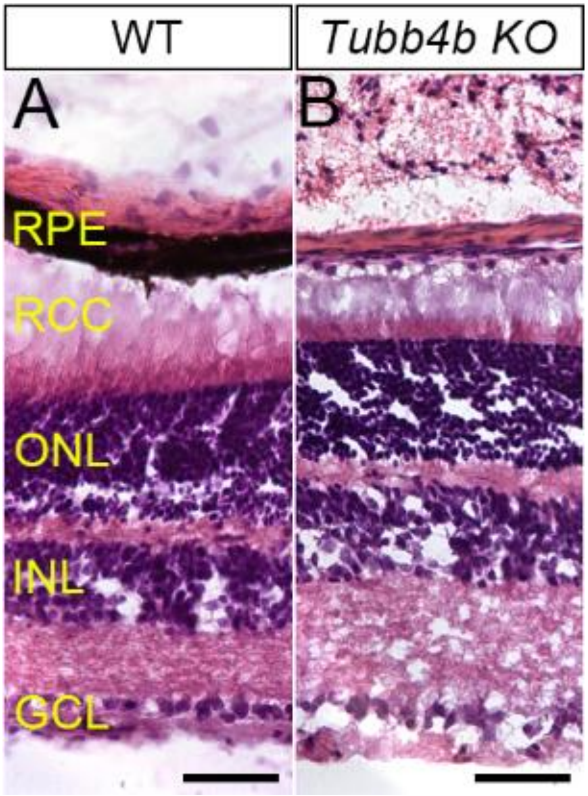

Fig. S5. *Tubb4bKO* retinal analysis. H&E staining of (A) WT retina (agouti animal) and (B) *Tubb4b KO* retina (albino animal) at P15. Scale=50µm. RPE=Retinal Pigmented Epithelium, RCC=Rod and Cone cells, ONL=Outer Nuclear Layer, INL=Inner Nuclear Layer, GCL=Ganglion Cell Layer.

Table S1. Genotypes of *Tubb4b* animals in litters at different ages

Numbers of animals of WT, heterozygous *Tubb4b* and homozygous *Tubb4b* animals at postnatal ages P0-P3 (14 litters), P4-P6 (9 litters), P7-P10 (18 litters).

Litter age	WT	Het	Mut
P0-P3	29	43	23
P4-P6	21	25	7
P7-P10	24	50	9

Development • Supplementary information
